# OverCOVID: an integrative web portal for SARS-CoV-2 bioinformatics resources

**DOI:** 10.1515/jib-2020-0046

**Published:** 2021-03-19

**Authors:** Md. Asif Ahsan, Yongjing Liu, Cong Feng, Ralf Hofestädt, Ming Chen

**Affiliations:** Department of Bioinformatics, College of Life Sciences, Zhejiang University, Hangzhou, 310058, China; Bielefeld University, Faculty of Technology, Bioinformatics and Medical Informatics Department, Bielefeld, Germany

**Keywords:** bioinformatics resources, coronavirus, COVID-19, SARS-CoV-2

## Abstract

Outbreaks of COVID-19 caused by the novel coronavirus SARS-CoV-2 is still a threat to global human health. In order to understand the biology of SARS-CoV-2 and developing drug against COVID-19, a vast amount of genomic, proteomic, interatomic, and clinical data is being generated, and the bioinformatics researchers produced databases, webservers and tools to gather those publicly available data and provide an opportunity of analyzing such data. However, these bioinformatics resources are scattered and researchers need to find them from different resources discretely. To facilitate researchers in finding the resources in one frame, we have developed an integrated web portal called OverCOVID (http://bis.zju.edu.cn/overcovid/). The publicly available webservers, databases and tools associated with SARS-CoV-2 have been incorporated in the resource page. In addition, a network view of the resources is provided to display the scope of the research. Other information like SARS-CoV-2 strains is visualized and various layers of interaction resources is listed in distinct pages of the web portal. As an integrative web portal, the OverCOVID will help the scientist to search the resources and accelerate the clinical research of SARS-CoV-2.

## 1 Introduction

**Figure 1:**
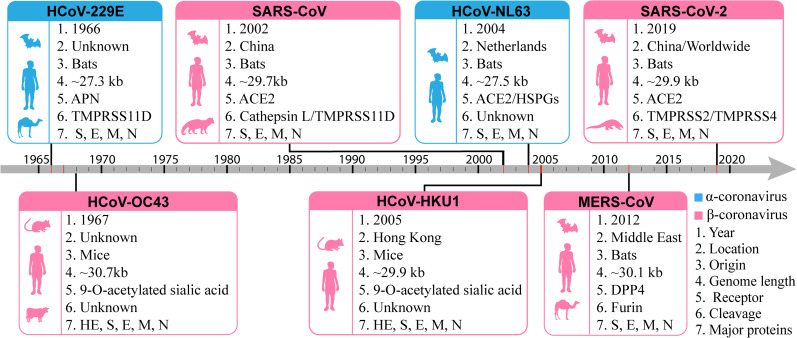
Timeline and basic properties of seven human coronaviruses. Two blue panels list alpha-coronavirus and the five pink panels list beta-coronavirus. All the boxes summarize the year of identification, location of first reported case, origin or natural host of the virus, genome length of virus, receptor, cleavage of the receptor, and major proteins of the respective human coronavirus. The left parts of the boxes present the main hosts for the corresponding coronaviruses. Two beta-coronaviruses, HCoV-OC43 and HCoV-HKU1 originate in mice and belong to hemagglutinin-esterase (HE) structural protein along with S, E, M and N. Other five viruses originated in bats and have only four major proteins S, E, M and N.

Since December 2019, the outbreak of severe acute respiratory syndrome coronavirus 2 (SARS-CoV-2) has spread across all over the world, posing a serious threat to global healthcare and society. The virus causes the pandemic coronavirus disease 2019 (COVID-19) and it has a high nucleotide sequence homology with SARS-CoV [1]. SARS-CoV-2 is the seventh coronavirus that has ability to infect humans and it belongs to the genus betacoronavirus of the coronavirinae subfamily (Figure 1). The Wuhan-Hu-1 reference genome sequence of SARS-CoV-2 (accession number: NC_045512) is a 29,903 bp single-stranded RNA (ss-RNA) and the genome encodes four main structural proteins: spike (S), envelope (E), membrane (M), and nucleocapsid (N), in which the surface unit S1 of the S protein can bind to angiotensin-converting enzyme 2 (ACE2) for attachment and cell entry. Owing to the ACE2 protein expression in human lung tissues [2], the SARS-CoV-2 infection causes severe pneumonia. Furthermore, ACE2 is also highly expressed in the kidney, heart and blood vessels [3], indicating a relationship between SARS-CoV-2 infection and the cardiovascular and renal system and causes kidney injury, heart failure, myocarditis and thrombosis [4]. Besides several other symptoms [5], including headache, dizziness, fatigue, muscle or body aches, sore throat, vomiting, diarrhea, loss of taste or smell [6], abdominal pain and vascular skin symptoms [7] are subsequently uncovered. Compared with SARS and MERS, the virion SARS-CoV-2 is proven more infectious. According to the statistics provided by WHO, as of 27 January 2021, globally, ∼99.63 million people have been infected by COVID-19, including 2,141,468 deaths.

To defeat COVID-19, previously developed techniques and technologies, such as next-generation sequencing, are being used [8]. Identification and characterization of the mutations could help to discover drug-resistance phenotype. Mutational evaluation of SARS-CoV-2 genomes has effectively been tracked by combining various clustering and phylogeny analysis [9]. It is also important to identify the zoonotic origin of the virus, that could help to monitor animals with high risk and preventing potential outbreaks. Computational pipeline has been developed to model the binding affinity between the SARS-CoV-2 spike receptor-binding domain (S-RBD) and ACE2 receptor of various intermediate hosts (vertebrate species) [10]. Computational drug repurposing studies have been applied for identifying fast drug treatment against COVID-19 by using the map of functional interactions between the proteins of virus and host [11]. These interactomics or network-based approaches have collected data on the virus–host protein–protein interactions (PPI), human PPIs, and the interaction between drugs and proteins where the drugs are targeted for both virus and human proteins [12].

Subsequent to the first release of the SARS-CoV-2 genome sequence on January 10th, 2020, many research groups focus their research on COVID-19 disease for characterizing the genome of the virus, identifying the pathways as well as the relationship between the virus and human host, trying to find the repurposable drug and developing vaccine against the pandemic. By the end of November, more than 175,000 SARS-CoV-2 genome sequences had been uploaded to GISAID [13], an open-access database that tracks viral evolution and spread worldwide. Researchers around the world are also sharing various types of SARS-CoV-2 related data, producing database to accumulate the data and developing tools for analyzing the data. Recently, we have developed an integrated web portal called OverCOVID by incorporating various bioinformatics resources related to COVID-19 [14]. It has mainly incorporated the publicly available web servers, databases and tools; listed the resources of various kind of interaction data and provided information on SARS-CoV-2 strain. The web portal could help the bioinformaticians to find useful data and information and accelerate the clinical research of SARS-CoV-2.

## 2 Historical overview of human coronaviruses

Coronaviruses (CoVs) are a large group of enveloped single-stranded positive-sense RNA viruses, causing mild to severe respiratory disease, including fever, common cold, pneumonia and bronchiolitis. Based on the similarity of genome sequence and structure, the coronaviruses are classified into four genera: alpha-, beta-, gamma- and delta-CoV [15]. The alpha- and beta-CoV family usually infect mammals and humans, whereas the gamma- and delta-CoV family generally infects birds. From the mid-1960s to the present, seven coronaviruses have been recognized to infect and cause disease in humans (Figure 1). Of the seven human coronaviruses (HCoVs), two HCoVs (229E and NL63) belong to the alpha-CoV genus, and the other five (OC43, SARS, HKU1, MERS and SARS- 2) belong to the beta-CoV genus. Bats are considered the natural hosts for most of the HCoVs [16], whereas only HCoV-OC43 and HCoV-HKU1 originated in mice [17]. Notably, the HCoVs originated in bats hold four major structural proteins (S, E, M and N); while in mice originated HCoVs, one more structural protein, hemagglutinin-esterase (HE), is observed along with the four proteins.

For entering in the host cell and development of virus infection, different human proteins or enzymes serve as receptors (Figure 1). For example, angiotensin-converting enzyme 2 (ACE2) has been identified as a significant entry receptor of human coronaviruses SARS-CoV [18] and SARS-CoV-2 [19], as well as HCoV-NL63 [20]. The aminopeptidase N (APN) and dipeptidyl peptidase 4 (DPP4) were discovered as an entry receptor for HCoV-229E and MERS-CoV, respectively, while mice originated beta-CoV HCoV-OC43 and HCoV-HKU1 use 9-O-acetylated sialic acid as a viral receptor [17].

The first human coronavirus, HCoV-229E, was identified in 1966. In the following year, another HCoV named HCoV-OC43 had emerged [21]. After a long gap, in November 2002, SARS-CoV first appeared in Guangdong province of China, and the next year, the virus spread to more than 25 countries of four continents (Asia, Europe, North America and South America) [22]. According to WHO, SARS has had a total of 8,096 diagnosed cases and 774 deaths. Since 2004, no confirmed cases of SARS reported anywhere in the world. In the same decade, two more HCoVs, NL63 and HKU1 were appeared in the Netherlands (2004) and Hong Kong (2005), respectively [23]. In June 2012, a highly pathogenic HCoV, MERS, was emerged in the middle east and as of 31 May 2020, it has caused a total of 2,562 confirmed cases with 34.4% fatality rate (881 deaths), the majority in Saudi Arabia. Until now, the virus is still infecting human. During 1 April and 31 May 2020, nine new cases were reported, including five deaths. SARS-CoV and the MERS-CoV studies have contributed the majority of current knowledge concerning the biological properties of coronaviruses, including the pathogenic mechanisms, functions of vital proteins, potential drug targets and treatment strategies. Also, the two previous disease outbreaks have provided valuable lessons about public health emergency response. These accumulated data and knowledge will shorten the path to effective treatments.

## 3 Materials and methods

### 3.1 Data compilation

Various information like resource names, categories, keywords, corresponding links and publications of the COVID-19 related resources were collected manually through online. Each resource was subjected to one or more of the following three categories: database, webserver and tool. The keywords are manually extracted from literature and web page descriptions. The OverCOVID web portal will track newly released resources and update regularly. The sequence data for visualizing the strains of SARS-CoV-2 was collected from GISAID (https://www.gisaid.org/CoV2020/).

### 3.2 Web portal construction

The OverCOVID web portal has been implemented using PHP 5.4.16 and bootstrap 3. MySQL 5.5.37 was used for data storage and efficient management. The visualization of the SARS-CoV-2 strains were performed using R programming language. Cytoscape 3.7.1 [24] was used to visualize the network of the resources and JavaScript library jQuery was used to make the network interactive or clickable to link with the corresponding resources pages.

## 4 Results and discussion

**Figure 2:**
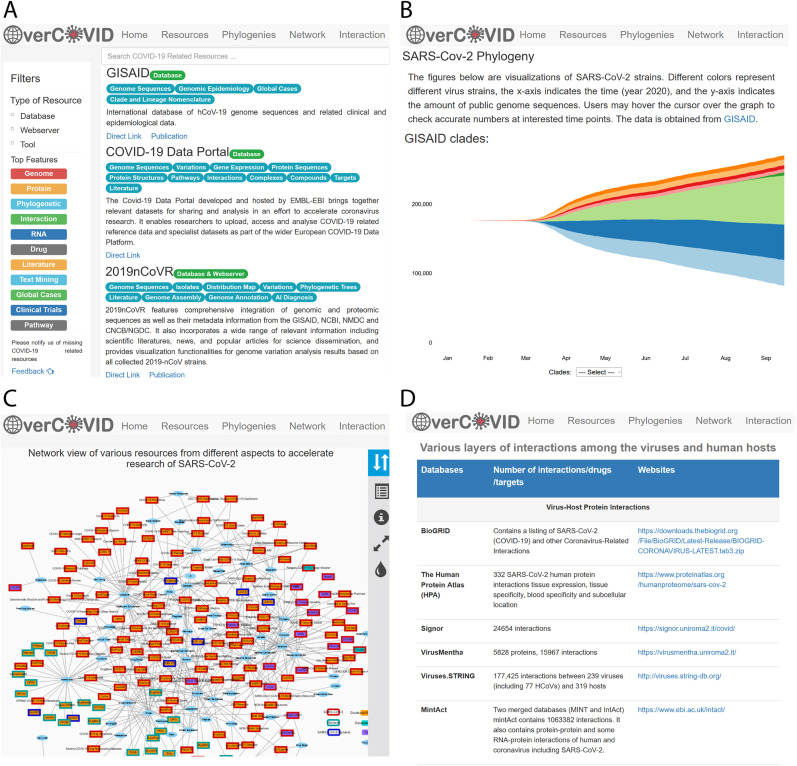
Overview of the OverCOVID web portal. (A) Resources page contains SARS-CoV-2 related online resources with resource name, resource type, features of the resources, brief description, URL of the resources and publication link. It facilitates users to search the desire resources by typing the resource name or the keywords in the search box. (B) SARS-CoV-2 phylogeny. It uses the sequence data from GISAID and shows the development of each strain of the virus since the beginning of the spread of the virus. It also provides a comparison of different nomenclature systems, including the marker gene variants representing for each virus strain. (C) Network view of various resources from different aspects to accelerate research of SARS-CoV-2. An additional network view of the resources is provided to visually indicate the scope of research for each database/tool and to intuitively present extra information. (D) Resources of various layers of interactions among the viruses and human hosts. The interaction resources page provides information on the resources for each type of molecular interaction as well as the corresponding link of the source site.

The OverCOVID web portal incorporates the resources associated with SARS-CoV-2 to provide easy access to various information that may be utilized in bioinformatics approaches and may contribute to the research of COVID-19. OverCOVID web portal is available at http://bis.zju.edu.cn/overcovid/. The included diverse information is allocated in multiple pages – ‘resources’, ‘phylogenies’, ‘network’ and ‘interaction’ – representing different aspects (Figure 2). These pages can be accessed by clicking the corresponding buttons in the top navigation bar.

**Figure 3:**
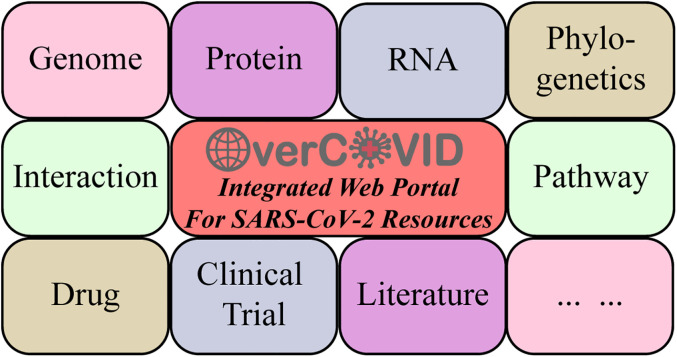
Bioinformatics resources and information on SARS-CoV-2 in the OverCOVID web portal.

### 4.1 Resources

In accordance with the types of resources, the OverCOVID grouped the resources into three classes – ‘web server’, ‘database’ and ‘tools’ in the resource page, and selecting a ‘filters’ option from the left sidebar provides a good starting point to search the resources (Figure 2A). For each page, five resources are viewed with resource name, resource type (on the green box), features of the resource (on cyan box), a short description, direct link and link of the publication (if have). Users can also click on ‘previous’ and ‘next’ buttons to move to other pages. Furthermore, on the basis of main features of the resources of the webservers, databases and tools associated with SARS-CoV-2 as well as COVID-19 could also be categorized into several classes such as genome, protein, RNA, phylogenetics, interaction, drug, literature, clinical trial, pathway and so on (Figure 3). These top features are listed in the left sidebar and clicking on the feature the resources would be shorted on the list. Moreover, these main features could be separated into many other keywords or sub-features (Table 1). Users can use these keywords in the search box of this page to find the desired resources.

**Table 1:** Top features, the corresponding keywords and some resources in the OverCOVID web portal.

Main features	Sub-features or keywords	Webserver/database/tools
Genome	Sequences, annotation, visualization, browser, assembly, sequencing protocols, variation, quality control, haplotype, isolates, mutation, gene expression, subtyping, gene ontology, genomic epidemiology, functional domain, primer design.	GISAID, nextstrain, covidex, GESS, pangolin, COVID-19 genotyping tool, SARS-CoV-2 alignment screen, UCSC SARS-CoV-2 genome browser, 2019nCoVR, CoV2ID, COVID-19 data portal, NCBI nucleotide sequences, SARS-CoV-2 data hub, ViPR SARS-CoV-2, WOLFRAM
Protein	Protein sequence, protein structure, PDB protein structures, protein expression, protein–protein interactions (PPI), function, localization, protein mutations, protein alignment, protein domains.	CoViProteins, coronavirus3D, neXtProt, COVID-19 docking server, COVID-19 molecular structure and therapeutics hub, COVID-19 UniProtKB, COVIDep, NCBI protein sequences, PROSITE, PubChem COVID-19 data, STRING COVID-19 host-interactome, SWISS-MODEL, the human protein atlas, VBRC, ViPR SARS-CoV-2, Virus-CKB
RNA	RNA-seq, RNA secondary structures, conservation, RNA motifs, RNA structures.	Rfam COVID-19 resources, VIRify
Phylogenetic	Phylogenetic trees, phylogenetic assignment, phylogenetic reconstructions, phylogenetic expectation.	Nextstrain, covidex, pangolin COVID-19 lineage assigner, 2019nCoVR, SARS-CoV-2 alignment screen, 2019nCoVR, CoV-GLUE, coronavirus typing tool, phylomeDB coronavirus phylomes, SARS-CoV-2 analysis workflow
Interaction	Drug interactions, protein–protein interactions (PPI), virus–host interactions, pathogen–host interactions, target-ligand interactions, chemical interactions.	COVID-19 UniProtKB, CORDIT, CoVex, COVID-19 coronavirus project, COVID-19 disease map, IntAct, COVIDminer, COVID-19 KnetMiner, P-HIPSTer, STRING COVID-19 host-interactome, VirHostNet
Pathway	KEGG pathway, SARS-CoV-2 pathways, pathway figures, signaling pathways.	WikiPathways, COVID-19 pathway figures, CoV-Hipathia, CoV-hipathia, PubChem COVID-19 data
Drug	Drug targets, drug design, druggable interactome, drug discovery, drug interactions, repurposable drugs, antiviral drugs, clinical trials.	Chemical checker, CoViLigands, D3SIMILARITY, D3targets-2019-nCoV, DrugBank COVID-19 dashboard, MolAICal, the COVID-19 drug and gene set library, canSAR, CoVex, COVID-19 docking server, Virus-CKB, ClinicalTrials.gov
Literature	Literature collection, literature curation, literature searching, literature corpus, literature triage.	LitCovid, CORD-19, COVID literature dataset, COVID-19 literature review, COVID-19 Intelligent Insight, COVID-19 open access, COVID-19 research database, CovidTriage, COVID-19 research explorer, medRxiv

### 4.2 Phylogenies

This page incorporates the phylogenetic information of SARS-CoV-2 genome obtained from GISAID and visualizes the strains (Figure 2B). Different colors represent different virus strains, where the *x*-axis indicates the time (by month) and the *y*-axis shows the number of public genome sequences. Hovering the cursor over the graph reveals the genome sequences’ number at the specific time points for the particular clades or lineages. Clicking the option ‘select’ on the box also shows the clades or lineage of the sequences. Users can also find a table showing the clades and corresponding marker variations provided by the Nextstrain (https://nextstrain.org/sars-cov-2/).

### 4.3 Resources Network

In the ‘resources network’ page, an additional network view of the resources (Figure 2C) is provided to visually indicate the scope of research for each database/tool and to intuitively present extra information (e.g. node degree may indicate the research focus). Squares represent the tools or databases/web platforms and the ellipses show the features (main focus areas) of the corresponding databases/tools. An interactable version of this network is available at http://bis.zju.edu.cn/overcovid/network.html. Users may zoom in/out, drag and click on the nodes to obtain more information and a list of the features (for the selected resources) and resources (for the selected features) will be appeared. Users can also go directly to the corresponding page of the resources by simply clicking on the list.

### 4.4 Interaction resources

Limiting the transmission of SARS-CoV-2 and manage patients with the most severe cases of the COVID-19, it is crucial to identify potential targets and the corresponding drug candidates. Integration of large-scale interactome in a network, such as human PPIs, virus–host protein interactions and drug-target interactions, assist computational based identification of potential targets and drug candidates [12], [25]. Network and system medicine-oriented web tools or web platforms, for example, CoVex [26], CORDIT [27], COVIDep [28] have been constructed by integrating virus–host and drug-target interactions to recommend repurposable drug candidates. In the OverCOVID web portal a distinct interaction resources page has developed by including various layers of interactions among the human host and viruses for example, virus–host PPI, human PPI, ncRNA associated interaction, drug-target interaction and the side effects of the drug (Figure 2D). This page has drawn a list of the resources/database name, the statistics with a short description of various interactions and the URL of the corresponding resources page.

#### 4.4.1 Virus–host protein interactions

This section contains the resources of the interaction of protein between host and various, including HCoV as well as SARS-CoV-2. Databases such as, BioGRID, the human protein atlas (HPA) includes the experimentally validated virus–host PPIs [11]. Other resources like VirusMentha, Virus.STRING, VirusHostNet offer users to download virus–host and virus–virus protein interaction of various pathogen species, including SARS-CoV, MERS-CoV, HCoV-229E, influenza, herpes viruses, zika virus, dengue virus and so one.

#### 4.4.2 Human protein–protein interactions

Human proteins that interacted with the proteins in virus–host interaction are being used in the SARS-CoV-2 study [29]. Some publicly available resources such as BioGRID, HPRD, STRING, mentha are listed, which incorporates both experimentally determined and computationally predicted human PPIs.

#### 4.4.3 ncRNA-associated interactions

ncRNAs could be used as a biomarker in the clinical research of COVID-19 [30], [31]. The resources that incorporate ncRNA-associated virus–virus, virus–host, host–virus, host–host, RNA-compound interactions, ncRNA–disease associations are listed in this section.

#### 4.4.4 Drug-target or drug-protein or drug-gene interactions

In the network-based drug repurposing studies the interactions of drug-targets are being used [26], [32]. The databases like DrugBank, BindingDB, STITCH has curated the interactions of the drug with gene or protein and are listed in this section.

#### 4.4.5 Drug side effects

Drug-induced side effects are also being assessed in the current network-based framework of COVID-19 studies [33]. Resources for drug side effects such as SIDER, DrugMatrix, DPIS are listed in the OverCOVID web portal.

## 5 Conclusion

Researchers and scientists have responded quickly to the COVID-19 pandemic by rapidly sequencing the virus genome, developing a testing kit to detect patients affected by SARS-CoV-2 and identify the repurposable drug to treat the patients. Bioinformaticians and data scientists worldwide have also promptly started collecting different dimensional data like epidemiological, clinical and biological data and developing webserver, database and tool for accumulating the data and offering the researcher for vast analysis. To make accessible these publicly available resources in one frame, we have developed an integrative web portal called OverCOVID. To our knowledge, this is the first of its kind in the web portal for SARS-CoV-2. We believe this web portal will be very helpful to the bioinformaticians and the researchers to find useful data and information from the resources and facilitate harnessing research of SARS-CoV-2.
